# 240‐week entecavir maleate treatment in Chinese chronic hepatitis B predominantly genotype B or C

**DOI:** 10.1111/jvh.13724

**Published:** 2022-07-06

**Authors:** Jing‐Hang Xu, Ya‐Nan Fan, Yan‐Yan Yu, Chong‐Wen Si, Zheng Zeng, Zhong‐Nan Xu, Jun Li, Qing Mao, Da‐Zhi Zhang, Hong Tang, Ji‐Fang Sheng, Xin‐Yue Chen, Qin Ning, Guang‐Feng Shi, Qing Xie, Xi‐Quan Zhang, Jun Dai

**Affiliations:** ^1^ Department of Infectious Diseases, Center for Liver Diseases Peking University First Hospital Beijing China; ^2^ Jiangsu Chia‐tai Tianqing Pharmaceutical Co, Ltd Nanjing China; ^3^ Department of Infectious Diseases The First Affiliated Hospital of Nanjing Medical University Nanjing China; ^4^ Department of Infectious Diseases Southwest China Hospital Chongqing China; ^5^ Department of Infectious Diseases The Second Affiliated Hospital of Chongqing Medical University Chongqing China; ^6^ Department of Infectious Diseases West China Hospital Chengdu China; ^7^ Department of Infectious Diseases The First Affiliated Hospital of Zhejiang University Hangzhou China; ^8^ Department of International Medicine, Beijing Youan Hospital Capital Medical University Beijing China; ^9^ Department and Institute of Infectious Diseases, Tongji Hospital, Tongji Medical College Huazhong University of Science and Technology Wuhan China; ^10^ Department of Infectious Diseases, Huashan Hospital Fudan University Shanghai China; ^11^ Department of Infectious Diseases, Ruijin Hospital Jiaotong University School of Medicine Shanghai China

**Keywords:** efficacy, entecavir, entecavir maleate, hepatitis B, chronic, safety

## Abstract

This study aimed to evaluate the efficacy and safety of entecavir(ETV) versus ETV maleate in Chinese patients with chronic hepatitis B(CHB). This was a randomized, double‐blind, double‐dummy, controlled, multicentre study. Patients were randomly assigned to receive 48 weeks of treatment with 0.5 mg/day ETV (group A) or 0.5 mg/day ETV maleate (group B), then, all patients received treatment with 0.5 mg/day ETV maleate from week 49 onwards. Patients were regularly followed up. Serum hepatitis B virus (HBV) markers were detected. Adverse events (AE) were recorded. The primary endpoint was the decline in HBV DNA in each group at the end of treatment. Secondary endpoints included the rate of HBV DNA below the lower limit of detection (LLOD) (20 I U/ml) at the end of treatment, the rate of hepatitis B e antigen (HBeAg) loss, the rate of HBeAg seroconversion and serum alanine aminotransferase (ALT) normalization. One hundred and thirty‐seven (71 in group A) patients with HBeAg‐positive CHB and 46 (21 in group A) patients with HBeAg‐negative CHB completed the 240‐week treatment and follow‐up. Baseline characteristics were well balanced between the two groups. For the HBeAg‐positive CHB patients, the mean HBV DNA level had similarly decreased from baseline in both groups (A: by 6.67 log_10_ IU/ml vs. B: by 6.74 log_10_ IU/ml; *p* > .05) at Week 240. Patients who achieved undetectable levels of serum HBV DNA (<20 IU/ml) at Week 240 were similar between groups (A:91.55% vs. B:87.88%; *p* > .05). Both groups achieved similar HBeAg seroconversion rates at week 240 (A:26.98% vs. B:20.97%; *p* > .05). Both groups achieved similar normalization of ALT (A:87.32% vs. B:83.61%; *p* > .05) at Week 240 (*p* > .05). For the HBeAg‐negative CHB patients, the mean HBV DNA level had similarly decreased from baseline in both groups (A: by 6.05 log_10_ IU/ml vs. B: by 6.10 log_10_ IU/ml; *p* > .05) at Week 240. Patients who achieved undetectable levels of serum HBV DNA at Week 240 were similar between groups (A:100% vs. B:100%). Both groups achieved similar normalization rates (A:90.91% vs. B: 95.45%; *p* > .05) of ALT at Week 240 (*p* > .05). In conclusion, long‐term ETV maleate treatment was safe and efficient in Chinese CHB predominantly of genotype B or C.

**Abbreviations**
AE: adverse event
ALT: alanine transaminase
anti‐HBe: hepatitis B e antibody
AR: adverse reaction
CHB: chronic hepatitis B
ETV: entecavir
FAS: full analysis set
HBeAg: hepatitis B e antigen
HBV: hepatitis B virus
HCC: hepatocellular carcinoma
LLOD: lower limit of detection
LOCF: last observation carry‐forward
M: median
NA: nucleos(t)ide analogues
PCR: polymerase chain reaction
Q: interquartile range
SAE: serious adverse event
SD: standard deviation
TAF: tenofovir alafenamide
TDF: tenofovir
ULN: upper limit of normal

## 1 INTRODUCTION

Hepatitis B virus (HBV) infection can lead to chronic liver diseases such as cirrhosis and liver cancer. Chronic HBV infection is still a substantial health burden in China, with 70 million people chronically infected by HBV.^1^, ^2^ Antiviral therapy is the key treatment of chronic HBV infection, which can inhibit the replication of HBV leading to the reduction of liver damage.^3^ Whenever indicated, standardized antiviral therapy should be initiated to treat chronic HBV infection.^4^ Entecavir (ETV) is one of the first‐line nucleos(t)ide analogues (NA) in the treatment of HBV.^4^

Although the efficacy and safety of long‐term ETV therapy have been reported previously, the limitations of those reports make it necessary to investigate the long‐term use of ETV at the dose of 0.5 mg daily in genotypes B and C chronic HBV infection.^5^ ETV maleate, a derivative of ETV, has been approved by The State Food and Drug Administration of China. Previous reports showed that 144 weeks of ETV maleate was effective and safe in Chinese patients predominantly infected with HBV genotype B or C.^5^, ^6^, ^7^, ^8^ Herein, we update the virological, serological and biochemical outcomes up to 240 weeks of ETV maleate in Chinese patients predominantly infected with HBV genotype B or C.

## 2 MATERIALS AND METHODS

### 2.1 Study population

The characteristics of the study population have been reported previously.^5^ Outpatients with chronic hepatitis B (CHB) were included at 10 sites located in Beijing, Shanghai, Chengdu, Chongqing, Nanjing and Hangzhou, China. Eligibility Criteria were as follows^5^:18–65 years of age, HBsAg‐positive for at least 6 months, HBV DNA ≥2 × 10^4^ IU/ml for those with positive hepatitis B e antigen (HBeAg) or HBV DNA ≥2 × 10^3^ IU/ml for those with negative HBeAg, alanine transaminase (ALT) ≥1.3 and <10 × upper limit of normal (ULN) and serum total bilirubin ≤2.5 × ULN. Patients with evidence of liver decompensation, hepatocellular carcinoma, co‐infection or super‐infection with other viruses, such as hepatitis A virus, hepatitis C virus, hepatitis delta virus, hepatitis E virus, Epstein–Barr virus or cytomegalovirus, were excluded.

### 2.2 Study design

The design of this two‐stage study design has been reported previously.^5^, ^6^, ^7^, ^8^ In stage 1, a randomized, double‐blind, double‐dummy, controlled, multicentre trial was carried out. The subjects were randomized (1:1) to the ETV group (0. 5 mg/day) (group A) or ETV maleate (0. 5 mg/day) (group B) group, receiving ETV or ETV maleate for 48 weeks. In stage 2, an open‐label study, all subjects received ETV maleate alone (0. 5 mg/day) from Week 49 through Week 240. At Week 100, adefovir dipivoxil (ADV) (10 mg/day) was added in subjects who experienced viral breakthrough, ETV resistance or coexistence of ALT increase and HBV DNA increase at Week 96. Resistance to ETV was defined as an additional substitution (rtI169 or rtT184, rtS202 or rtM250) on the base of pre‐existing lamivudine‐associated resistance substitutions (rtM204V with or without rtL180M). The subjects who had add‐on ADV were excluded from the efficacy analysis at Weeks 216 and 240.

The study was designed by the sponsor (Jiangsu Chia Tai Tianqing Pharmaceutical Company) in collaboration with primary investigators. The study was conducted in accordance with the guidelines of the Declaration of Helsinki and the principles of Good Clinical Practice and was approved by the Ethics Committee of Peking University First Hospital. All patients signed written informed consent. This trial was registered with ClinicalTrials.gov (NCT01926288).

### 2.3 Efficacy endpoints

The primary endpoint was the decline in HBV DNA from baseline in each group at the end of treatment. Secondary endpoints included the rate of HBV DNA below the lower detection limit (20 I U/ml), the rate of HBeAg loss, the rate of HBeAg seroconversion and serum alanine aminotransferase (ALT) normalization at the end of treatment.

### 2.4 Safety analysis

During the study, we detected the blood routines, urine routines, blood urea nitrogen and serum creatinine regularly at every visit. Abdominal sonography and alpha‐fetoprotein were detected every 48 weeks. Adverse events (AEs) were recorded at every visit.

### 2.5 Laboratory tests

The Viral Laboratory of Peking University First Hospital was put in charge of the Virological and serological tests. HBV serological markers (HBsAg, anti‐HBs, HBeAg and anti‐HBe) were detected by commercially available chemiluminescence immunoassays (Abbott Laboratories). Serum HBV DNA was tested by Roche Cobas Ampliprep/Cobas Taqman™ polymerase chain reaction (PCR) assay that reached a lower limit of detection (LLOD) of 20 IU/mL. Genotyping and resistance analysis were completed by direct sequencing PCR.^9^

### 2.6 Statistical analysis

Hepatitis B virus DNA levels were logarithmically transformed for analysis. Continuous variables were expressed as mean with standard deviation (SD) or median (M) with interquartile range (Q) and were compared by *t*‐tests or Wilcoxon rank sum test where appropriate. Chi‐square test or Fisher's exact test was used for comparison of categorial data. We used SPSS (Statistical Package for the Social Sciences) ver. 17.0 (SPSS) to perform statistical analysis. All statistical tests used a bilateral test. Two‐tailed *p* value of < .05 was considered statistically significant.

## 3 RESULTS

### 3.1 Study population and Baseline characteristics

In total, 279 patients were randomized and received at least one dose of study drug.^5^ Four of 279 patients were excluded from the full analysis set (FAS) because of the lower HBV DNA level than the inclusion criteria. Accordingly, 275 patients were included in FAS. At baseline, the two groups were well balanced.^5^ A total of 50 patients discontinued therapy before Week 144 which was detailed previously.^5^ An extra 42 patients were lost to follow‐up from Week 145 through Week 240. Consequently, a total of 183 patients completed the 240‐week treatment and follow‐up.

### 3.2 Virological endpoints

One hundred and thirty‐seven (71 in group A) patients with HBeAg‐ positive CHB and 46 (21 in group A) patients with HBeAg‐negative CHB completed the 240‐week treatment and follow‐up (Table 1).

**TABLE 1:** Reduction of HBV DNA levels from baseline in CHB patients at Weeks 216 and 240 (Mean ± SD, log_10_IU/ml)

Group	HBeAg‐positive		HBeAg‐negative	
	216 w	240 w	216 w	240 w
A	6.65 ± 1.41	6.67 ± 1.77	6.06 ± 1.31	6.05 ± 1.26
B	6.66 ± 1.11	6.74 ± 1.60	6.06 ± 1.23	6.10 ± 1.53
*p*	.959	.803	.996	.912

Abbreviations: CHB, chronic hepatitis B; HBeAg, hepatitis B e antigen; HBV, hepatitis B virus; SD, standard deviation.

For HBeAg‐positive patients, the mean reduction of serum HBV DNA levels from baseline to Week 216(A: by 6.65 vs. B: by 6.66 log_10_IU/ml, *p* > .05) and 240 (A: by 6.67 vs. B: by 6.74 log_10_ IU/ml, *p* > .05) were similar in group A and B (Table 1). The HBV DNA undetectable rates were 91.55% in group A and 87.88% (*p* > .05) in group B at Week 240 (Table 2).

**TABLE 2:** Rate of undetectable HBV DNA (<20 IU/ml) in CHB patients at Weeks 216 and 240

Group	HBeAg‐positive CHB, No (%)		HBeAg‐negative CHB, No (%)	
	216 w	240 w	216 w	240 w
A	63 (81.82)	65 (91.55)	22 (95.65)	21 (100)
B	58 (84.06)	58 (87.88)	27 (100)	25 (100)
*p*	.827	.577	.460	.912

Abbreviations: CHB, chronic hepatitis B; HBeAg, hepatitis B e antigen; HBV, hepatitis B virus.

For HBeAg‐negative patients, the mean decline of HBV DNA was comparable at Weeks 216 (A: by 6.06 vs. B: 6.06 log_10_ IU/ml, *p* > .05) (Table 1) and 240 (A: by 6.05 vs. B: 6.10 log_10_ IU/ml, *p* > .05) (Table 2). The HBV DNA undetectable rates were comparable between the two groups (A: 100% vs. B: 100%) at Week 240 (Table 2).

### 3.3 Serologic endpoints

With the prolongation of treatment time, the serum HBeAg loss rate and HBeAg seroconversion rate in both groups showed an increasing trend. There was a statistical difference in HBeAg loss rates at Week 240 between groups A and B (54.41% vs 31.25%, *p* < .05). However, HBeAg seroconversion rates were not statistically different between the two groups of HBeAg‐positive patients (Table 3).

**TABLE 3:** HBeAg loss and seroconversion in HBeAg‐positive CHB patients at Weeks 216 and 240

Group	HBeAg loss, No (%)		HBeAg seroconversion, No (%)	
	216 w	240 w	216 w	240 w
A	39 (51.32)	37 (54.41)	17 (24.29)	17 (26.98)
B	19 (27.54)	20 (31.25)	13 (19.40)	13 (20.97)
*p*	.0041	.0086	.5396	.5308

Abbreviations: CHB, chronic hepatitis B; HBeAg, hepatitis B e antigen; HBV, hepatitis B virus.

### 3.4 Biochemical endpoints

The rates of ALT normalization through Week 240 are described in Table 4. For the HBeAg‐positive patients, both groups achieved similar normalization of ALT (A: 87.32% vs. B: 83.61%; *p* > .05) at Week 240 (*p* > .05) (Table 4). For the HBeAg‐negative patients, ALT normalization rates were comparable (A: 90.91% vs. B: 95.45%; *p* > .05) at Week 240 as well.

**TABLE 4:** ALT normalization rates in CHB patients at Weeks 216 and 240

Group	HBeAg‐positive CHB, No (%)		HBeAg‐negative CHB, No (%)	
	216 w	240 w	216 w	240 w
A	69 (90.79)	62 (87.32)	21 (91.30)	20 (90.91)
B	57 (82.61)	51 (83.61)	22 (95.65)	21 (95.45)
*p*	.2173	.6226	1.000	1.000

Abbreviations: ALT, alanine transaminase; CHB, chronic hepatitis B; HBeAg, hepatitis B e antigen; HBV, hepatitis B virus.

### 3.5 Resistance surveillance

None of the HBeAg ‐ negative CHB patients developed ETV resistance through Week 240. For HBeAg‐positive CHB patients, seven patients developed resistance at Week 144. Only 2 of 7 were NA‐naive and received ETV maleate monotherapy, which resulted in an ETV resistance rate of 1.16% in NA‐naïve HBeAg‐positive CHB at Week 144.^5^ Mutations of the reverse transcriptase region were detected in 29 samples of 21 subjects with HBV DNA greater than 52 IU/ml at Weeks 192 and 240 including those who were excluded in the efficacy analysis due to add‐on ADV therapy or baseline resistance to lamivudine or ADV. The mutation profiles were obtained in 26 of the 29 patients. ETV resistance (rt L180M, rtM204V, rtM250L) was confirmed only in one subject at Week 240 who had a HBV DNA level of 6.1 log_10_ IU/ml. At Week 192, this subject possessed a mutation pattern of lamivudine resistance (rt L180M, rtM204V) and a HBV DNA level of 3.26 log_10_ IU/ml.

### 3.6 Safety analysis

In total, 279 patients were included in the safety analysis set. Safety reports for the first 144 weeks of the study have been published.^5^ From Week 145 through Week 240, the frequency of AE was similar in both groups (12.6% in group A and 16.0% in group B, *p* > .05). Overall, 2 and 4 cases of adverse reaction (AR), all of which were mild, occurred in group A and group B (*p* > .05), respectively. AR included a mild increase in ALT, increase in total bilirubin, abdominal pain, viral relapse due to discontinuation of study drug.

Three and 2 cases of SAE occurred in group A and group B (*p* > .05), respectively. Subjects 31 and 41 in group A received an induced abortion to terminate an unwanted pregnancy. The spouses of subject 44 in group A, subjects 36 and 228 in group B got pregnant. None of the other SAEs was judged to be related to the study drug. No discontinuation due to AE or AR occurred. No liver cancer or death occurred.

## 4 DISCUSSION

Given the long duration of ETV, and other NAs, in CHB patients, the long‐term data on ETV are of great concern. Although the efficacy and safety of ETV in CHB patients has been reported previously, ensuring ETV as one of the first‐line anti‐HBV drugs worldwide,^10^, ^11^ the long‐term data from large prospective studies in NA‐naive CHB patients with genotypes B or C treated with ETV 0.5 mg daily are insufficient.^5^

ETV maleate is a derivative of ETV, showing similar efficacy and safety in previous reports.^5^, ^6^, ^7^ Based on the data of phase III clinical trials,^5^, ^6^, ^7^ETV maleate has been approved by the State Food and Drug Administration of China. More importantly, with the advantage of lower price, ETV maleate has been widely used in China. Previously, we reported the data of 144 week ETV maleate treatment in Chinese CHB patients predominantly (98.5%) infected with genotype B or C, which showed excellent viral suppression with 66.67% of HBeAg‐positive and 100% of HBeAg‐negative patients achieving undetectable HBV DNA levels (<20 IU/ml), respectively. Only 1.16% NA‐naïve HBeAg‐positive and zero HBeAg‐negative CHB patients developed ETV resistance, and no clinical serious AR was found after 3 years of treatment.^5^

In this study, we presented the updated data of the clinical trial through Week 240. In terms of virologic endpoints, 240‐week treatment of ETV maleate led to the reduction of HBV DNA by 6.74 log_10_ IU/ml and 6.10 log_10_ IU/ml in HBeAg‐positive and HBeAg‐negative CHB patients, respectively. Undetectable HBV DNA rates were 87.88% and 100% in HBeAg‐positive and HBeAg‐negative CHB patients, respectively. For serologic endpoints, a 240‐week treatment of ETV maleate resulted in an HBeAg seroconversion rate of 20.97%. For biochemical endpoints, ALT normalization rates were 83.61% and 95.45% in HBeAg‐positive and HBeAg‐negative CHB patients, respectively. These results proved the potent anti‐HBV efficacy of ETV maleate in CHB patients with genotype B or C, which were consistent with the previous research on ETV in CHB predominantly genotypes A.^12^ Five years of ETV treatment led to 94% (88/94) HBeAg‐positive CHB patients achieving HBV DNA < 300 copies/ml (~52 IU/ml) and 80% (78/98) achieving ALT normalization.^12^ In addition to patients who obtained a serological response during the study of ETV‐022, 23% (33/141) achieved HBeAg seroconversion during the study of ETV‐901 and 1.4% (2/145) lost HBsAg in the fifth year. This is similar to the results of our study.

Regarding the comparison between the two groups, almost all the endpoints are comparable at all timepoints, thus indicating similar outcomes between groups and further proving the similarity between ETV and ETV maleate. However, the HBeAg loss rate is an exception. Group A (ETV for 48 weeks, then ETV maleate through week 240) had higher HBeAg loss rate than Group B (ETV maleate for 240 weeks). The difference may be due to the reappearance of HBeAg in 14 patients who had experienced HBeAg loss at Week 144. Given the fact that HBeAg seroconversion rate, the more important landmark in the control of HBV infection, was comparable between groups, the difference between HBeAg loss rate was not so significant. For clinical setting, this difference reminded us of the instability of HBeAg status in the early stage of HBeAg loss.

The present study had some positive features. First, it was the largest prospective multi‐centre study of ETV maleate treatment in China. Second, it adopted highly sensitive tests, the Cobas Taqman assay (LLOD 20 IU/ml), to measure serum HBV DNA levels. With 87.88% of HBeAg‐positive and 100% of HBeAg‐negative patients achieving undetectable HBV DNA levels at Week 240, it further verified that ETV maleate is a NA with extremely high antiviral potency. Third, approximately 70% patients completed the 240‐week follow‐up, which laid the foundation for the quality of the clinical trial.

However, there are some limitations. First, the sampling bias was inevitable. The subjects enrolled in this study were not able to represent the population of HBV patients. Therefore, we focused on the patients predominantly infected by genotype B and C, which is the characteristic of the subjects in this study. Second, histological outcome at Week 240 was not available because it's not in the follow‐up plan. Third, at the beginning of the study, we did not use FibroScan to measure liver fibrosis because the technology was new at that time. Future research may pay more attention to these issues. Finally, the number of HBeAg‐negative patients was relatively small. Only 57 patients were included in FAS in this study. However, the number had met the requirement of sample size for statistics.^5^ In addition, 46 (81.0%) HBeAg‐negative patients completed the 240‐week treatment.^12^

In conclusion, a 240‐week ETV maleate monotherapy was effective and safe in Chinese CHB patients. It showed potent HBV suppression, with 87.88% of HBeAg‐positive and 100% of HBeAg‐negative patients achieving undetectable HBV DNA. ETV resistance was rare in NA‐naive CHB patients. The safety profile of ETV maleate was promising.

## AUTHOR CONTRIBUTIONS

Yan‐Yan Yu, Chong‐Wen Si, Jun Dai, Xi‐Quan Zhang and Zhong‐Nan Xu designed the study. Jing‐Hang Xu, Zheng Zeng, Jun Li, Qing Mao, Da‐Zhi Zhang, Hong Tang, Ji‐Fang Sheng, Xin‐Yue Chen, Qin Ning, Guang‐Feng Shi, Qing Xie and Qing Xie collected data. Jing‐Hang Xu and Ya‐Nan Fan analysed and interpreted the data and wrote the manuscript. Yan‐Yan Yu approved the final manuscript. Chong‐Wen Si supervised the study. All authors had full access to the final version of the report and agreed to the submission.

## CONFLICT OF INTEREST

Qing Xie has acted as a consultant for Novartis, Bristol‐Myers Squibb and Roche. Guang‐Feng Shi has been a member of advisory committees or review panels, received consulting fees from Novartis, GlaxoSmithKline and Bristol‐Myers Squibb. Guang‐Feng Shi has acted as a consultant for Novartis, Bristol‐Myers Squibb, GlaxoSmithKline and Roche. Other authors have nothing to declare.

## CONSORT 2010 STATEMENT

The authors have read the CONSORT 2010 statement, and the manuscript was prepared and revised according to the CONSORT 2010 statement.

## Data Availability

The data that support the findings of this study are available from the corresponding author upon reasonable request.
